# Cortical atrophy and amyloid and tau deposition in Down syndrome: A longitudinal study

**DOI:** 10.1002/dad2.12288

**Published:** 2022-04-01

**Authors:** Concepcion Padilla, Victor Montal, Madeleine J. Walpert, Young T. Hong, Tim D. Fryer, Jonathan P. Coles, Franklin I. Aigbirhio, Sigan L. Hartley, Ann D. Cohen, Dana L Tudorascu, Bradley T. Christian, Benjamin L. Handen, William E. Klunk, Anthony J. Holland, Shahid H. Zaman

**Affiliations:** ^1^ Cambridge Intellectual and Developmental Disabilities Research Group, Department of Psychiatry University of Cambridge Cambridge UK; ^2^ Memory Unit and Biomedical Research Institute Sant Pau (IIB Sant Pau), Neurology Department Santa Creu and Sant Pau Hospital Barcelona Spain; ^3^ The Network Center for Biomedical Research in Neurodegenerative Diseases (CIBERNED) Madrid Spain; ^4^ Wolfson Brain Imaging Centre, Department of Clinical Neurosciences, Cambridge Biomedical Campus University of Cambridge Cambridge UK; ^5^ Division of Anaesthesia, Department of Medicine, Cambridge Biomedical Campus University of Cambridge Cambridge UK; ^6^ Waisman Center University of Wisconsin‐Madison Madison Wisconsin USA; ^7^ Department of Psychiatry University of Pittsburgh Pittsburgh Pennsylvania USA; ^8^ Cambridgeshire and Peterborough NHS Foundation Trust Fulbourn Hospital Cambridge UK

**Keywords:** Alzheimer's disease, amyloid deposition, cortical atrophy, Down syndrome, longitudinal, tau deposition

## Abstract

**Introduction**: The Down syndrome population has a high prevalence for dementia, often showing their first clinical symptoms in their 40s. **Methods**: In a longitudinal cohort, we investigate whether amyloid deposition at time point 1 (TP1) could predict cortical thickness change at time point 2 (TP2). The association between tau burden and cortical thickness was also examined at time point 3 (TP3). **Results**: Between TP1 and TP2 there was pronounced cortical thinning in temporo‐parietal cortices and cortical thickening in the frontal cortex. Baseline amyloid burden was strongly associated to cortical thinning progression, especially in the temporo‐parietal regions. At TP3, tau deposition negatively correlated with cortical atrophy in regions where tau usually accumulates at later Braak stages. **Discussion**: A higher amount of amyloid accumulation triggers a cascade of changes of disease‐causing processes that eventually lead to dementia. As expected, we found that regions where tau usually accumulates were those also displaying high levels of cortical atrophy.

## 1 INTRODUCTION

Most people with Down syndrome (DS) develop Alzheimer's disease (AD) pathology, showing the signs of dementia in their 40s and 50.^1^, ^2^ The presence of AD in DS is thought to be due to the presence of trisomy 21, which leads to an overproduction of the amyloid precursor protein gene product and an accumulation of the insoluble neurotoxic amyloid beta (Aβ),^3^ which is linked to the development of AD.^4^

Early in life, cerebrospinal fluid (CSF) Aß_42_ levels in DS are increased, becoming relatively normal as these individuals age, but later decreasing abnormally as they get older, representing deposition of Aß into plaques.^5^ Regarding amyloid deposition in the brain first becomes apparent in the striatum (although this finding is still uncertain, see Reference 6), extending to the frontal lobe, and then followed by the posterior areas, and finishing in the medial temporal lobe.^7^, ^8^, ^9^, ^10^ This pattern is similar with the familial, but not the sporadic forms of AD where striatal amyloid is not an early feature.^11^ After amyloid is accumulated, cortical thinning arises, extending from the lateral parieto‐temporo‐occipital cortex to the medial posterior cingulate and precuneus cortices.^5^, ^8^ The topography of cortical atrophy resembles the distribution seen typically in sporadic and familial autosomal dominant AD (ADAD).^11^

Most studies in the general population^12^, ^13^, ^14^ have shown that the progression of amyloid deposition correlates negatively with the spreading pattern of cortical thinning, especially at the earlier stages of the disease.^15^, ^16^ Nevertheless, the specific brain areas where amyloid builds up and the cortex gets thinner do not usually overlap, leading to the conclusion that amyloid is not the direct cause of cortical atrophy and that other processes, such as tau deposition, might be mediating the relationship between these two factors.^11^, ^13^ In fact, CSF phosphorylated tau increases with age and usually correlates with clinical symptoms.^5^

Cortical atrophy and abnormal levels of amyloid deposition can be observed in the brain approximately a decade before than the age of the dementia onset.^5^ It follows an order similar to what is found in autosomal and sporadic AD, except for the hippocampal volume, which is reduced in DS much earlier.^5^ Amyloid deposition in the brain seems to reach a plateau once the first clinical symptoms have been detected,^18^ both in DS and ADAD.

The implications of amyloid burden on cortical morphology in the long term have been little investigated in people with DS and AD. In a cross‐sectional study, Mak et al.^19^ found that the level of amyloid accumulation was highly correlated with cortical thinning across the brains of people with DS. When the pattern of regional amyloid deposition was compared with the regional cortical thickness, temporo‐parietal regions showed a negative correlation, indicating that in those specific areas both measures overlapped.

Currently, the literature (see eg, Ref 5, 11, and 20) supports the idea of neurodegeneration being driven by other factors such as the accumulation of neurofibrillary tangles in specific regions of the brain.

**HIGHLIGHTS**

- After 2 years, whole‐brain amyloid deposition did not change significantly.
- There was pronounced cortical atrophy in the temporo‐parietal cortices and thickening in the frontal cortex.
- Amyloid deposition at time point 1 (TP1) negatively correlated with the *change* in cortical thickness in temporal‐parietal regions.
- The rate of cortical thinning was explained by the level of amyloid deposition at TP1.
- Tau deposition was negatively correlated with cortical atrophy in regions where tau usually accumulates at Braak stages III, IV, and V in the non–Down syndrome (DS) population.

**RESEARCH IN CONTEXT**

1. **Systematic review**: The authors reviewed the literature using online databases looking for articles assessing change in cortical thickness as well as amyloid and tau deposition during the progression of the Alzheimer's disease (AD) in the Down syndrome (DS) population. The number of studies investigating the degree in which these biomarkers are associated and how they are co‐localized in the DS brain are scarce.
2. **Interpretation**: After 2 years, whole‐brain amyloid deposition did not change significantly, but there was pronounced cortical thinning in the temporo‐parietal cortices and thickening in the frontal cortex. The rate of cortical thinning was explained by the level of amyloid deposition at baseline. Tau deposition was negatively correlated with cortical thinning, showing that regions in the medial and basal temporal lobe that usually accumulate tau at later Braak stages also show cortical atrophy in the DS population.
3. **Future directions**: Our results support that tau deposition follows a progression pattern similar to that of familial and sporadic AD, and also co‐localizes with cortical atrophy. Patterns of cortical thickness and amyloid and tau deposition change should be explored, considering plasma and cerebrospinal fluid (CSF) biomarkers to explore how they interact along the course of the AD disease.

However, only the study of Rafii et al. ^21^ has explored the relationship between tau and cortical atrophy in DS. These authors compared tau Braak stage scores and structural magnetic resonance imaging (MRI) canonical variate scores, showing that only those DS patients revealing a cortical AD‐related pattern were the ones displaying abnormal levels of amyloid and tau deposition.

The study of the relationship between these neuroimaging biomarkers (ie, amyloid and tau positron emission tomography [PET] and structural MRI) is important as it can inform about the AD continuum in the DS population in a more objective manner.^5^ The National Institute on Aging and the Alzheimer's Association (NIA‐AA) proposed the AT(N) framework^22^ to objectively track the changes that occur along the AD progression from the preclinical to the latest stages of the disease. This biological definition of AD in people with DS will facilitate their inclusion and follow‐up in prospective preventive clinical trials.^23^

To our knowledge, only the present study has quantified the degree to which cortical atrophy, and amyloid and tau deposition are associated with each other in people with DS and has explored whether they are co‐localized. Using a longitudinal design, the aim of the present study was to investigate whether the level of amyloid burden found at time point 1 (TP1) correlated with cortical atrophy after ≈2 years (time point 2 [TP2]) in the entire brain and in specific regions in a sample of 11 adults with DS. In addition, at a third time point (TP3), ≈2 years after TP2, in eight subjects from the initial sample, we examined whether tau deposition was associated with cortical atrophy in specific regions of the brain.

We tested the following hypotheses: (1) the DS group will show significant cortical atrophy and amyloid accumulation over time; (2) levels of amyloid deposition at TP1 will be associated with cortical atrophy at TP2; and finally, (3) tau deposition will correlate negatively with cortical atrophy within specific brain regions where neurofibrillary tangles are typically found in the AD progression.^24^

## 2 MATERIALS AND METHODS

### 2.1 Study design and participants

Eleven participants took part in the study (see Table 1). These participants were part of the DS cohort reported in previous studies,^8^, ^9^ who wished to return for a second scan after 2.5 years. Eight of these participants came back after a further 2.5 years for a third set of scans, this time using a different scanner (GE Signa PET/MR). Trisomy 21 was confirmed via karyotyping. Participants underwent structural MRI and PiB positron emission tomography (PET) neuroimaging at TP1 and TP2 between the years 2012 and 2015, with the interval between TP1 and TP2 ranging between 19 and 36 months (mean 29.73 months). The participants who returned for TP3, undertook a third set of scans in 2017, consisting of structural MRI and AV‐1451 PET neuroimaging. TP3 was part of the baseline of the Neurodegeneration in Aging Down Syndrome (NiAD) study (see Ref 25 for further details). The scan interval between TP1 and TP3 ranged between 48 and 67 months (mean 58.89 months). All scans took place at the Wolfson Brain Imaging Centre at the University of Cambridge. Participants were also evaluated for the presence or not of dementia using the Cambridge Examination for Mental Disorders of Older People with Down's Syndrome and Other Intellectual Disabilities (CAMDEX‐DS) informant schedule,^26^ and an experienced psychiatrist made the dementia diagnosis. Written consent was obtained from all adults with DS with the capacity to consent. For participants lacking the capacity to consent, the procedures set out in the England and Wales Mental Capacity Act (2005) were followed. All the data were anonymized for data protection. The study was performed in accordance with The Code of Ethics of the World Medical Association (Declaration of Helsinki) for experiments involving humans and was approved by the National Research Ethics Committee of East of England and the Administration of Radioactive Substances Advisory Committee.

**TABLE 1:** Demographic data

Age			Interval	Gender		Diagnosis		
TP1	TP2	TP3	(Months)	Female	Male	No decline	Cognitive decline	Dementia
43.0 (34 – 51)	45.7 (36 – 53)	48.1 (38‐56)	TP1‐TP2: 29.7 (19 ‐ 36)	TP1‐TP2: 5	TP1‐TP2: 6	TP1: 8	TP1: 2	TP1:1
			TP1‐TP3: 58.4 (48‐67)	TP3: 3	TP3: 5	TP2:7	TP2:0	TP2:4
						TP3:4	TP3:2	TP3:2

Abbreviation: TP, Time point.

### 2.2 Neuroimaging data acquisition

#### 2.2.1 Magnetic resonance imaging

##### Time points 1 and 2

T1‐weighted magnetization‐prepared rapid gradient echo (T1‐MPRAGE) images were obtained with a Siemens Verio 3T MRI scanner (Siemens AG, Erlangen, Germany) scan using the following parameters: repetition time (TR) = 2300 milliseconds, echo time (TE) = 2.98 milliseconds, inversion time = 900 milliseconds, flip angle = 9°, 176 slices, and 1.0 × 1.0 × 1.0 mm^3^ voxel size. Receiver bandwidth and echo spacing were 240 Hz/pixel and 7.1 milliseconds, respectively. Total scan acquisition time was 9 minutes 14 seconds.

##### Time point 3

T1‐MPRAGE images were obtained with a GE Signa PET/MR scanner (GE Healthcare, Waukesha, WI, USA) using the following parameters: repetition time (TR) = 7.64 milliseconds, echo time (TE) = 3.08 milliseconds, inversion time = 400 milliseconds, flip angle = 11°, 196 slices, and a 1.055 × 1.055 × 1.20 mm^3^ voxel size. Receiver bandwidth and echo spacing were 31.25 Hz/pixel and 3.08 milliseconds, respectively.

#### 2.2.2 Positron emission tomography

##### Time points 1 and 2

[^11^C]PiB was produced with a radiochemical purity higher than 95% and a specific activity higher than 150 GBq/μmol. The radiotracer was injected as a bolus (median = 545 MBq, interquartile range = 465 to 576 MBq) through an antecubital venous catheter. A 15‐minute transmission scan using rotating Ge rod sources was applied to correct for photon attenuation previous to the PET scan. After that, dynamic [^11^C]PiB PET images were acquired in three‐dimensional (3D) mode on a GE Advance PET scanner 90 minutes post‐injection. Fifty‐eight frames were acquired (18 × 5 seconds, 6 × 15 seconds, 10 × 30 seconds, 7 × 1 minutes, 4 × 2.5 minutes, and 13 × 5 minutes). Sinogram data for each frame were reconstructed into a 128 × 128 × 35 image array with a voxel size of 2.34 × 2.34 × 4.25 mm^3^ using the PROMIS 3D filtered back projection algorithm.^27^ Random coincidences, normalization, attenuation, dead time, scatter, and sensitivity were corrected.

##### Time point 3

[^18^F]AV‐1451 PET and MRI data were acquired at the same time on the GE Signa PET/MR scanner, with PET data acquisition occurring 75 to 105 minutes post‐injection. The [^18^F]AV‐1451 list mode data were reconstructed into 6 × 5 minutes images using ordered subsets expectation maximization (OSEM)^28^; with six iterations, 16 subsets, and a voxel size of 2.0 × 2.0 × 2.8 mm^3^.

### 2.3 Neuroimaging data processing

T1‐MPRAGE images were processed using FreeSurfer v6.0 (http://surfer.nmr.mgh.harvard.edu/) to obtain cortical thickness measurements in two different formats: (1) whole‐brain vertex‐wise, and (2) region of interest (ROI) values. Details for surface reconstruction and estimation of cortical thickness have been detailed previously elsewhere.^28^, ^29^, ^30^ T1‐MPRAGE images from TP1 and TP2 were processed with the FreeSurfer longitudinal stream,^32^ which creates an unbiased within‐subject template using the structural images of both time points. Estimated surfaces were inspected and corrected for errors.^33^ Because T1‐MPRAGE images from the TP3 were acquired with a different scanner, they were not included in the longitudinal stream, but processed separately.

Cortical thickness values from each of the 34 ROIs per hemisphere in the Desikan‐Killiany atlas^34^ were extracted using FreeSurfer.

PET images within each dynamic series were realigned with Statistical Parametric Mapping 12 (Wellcome Trust Centre for Neuroimaging, University College London) and the resulting mean images were co‐registered to their corresponding T1‐MPRAGE image using FreeSurfer. PET images were merged with the default Freesurfer segmentations to facilitate PET‐MRI integration and partial‐volume correction.^35^ Co‐registration was checked for each participant. Partial‐volume corrected ROI data were derived using the geometric transfer matrix (GTM) technique provided by PetSurfer.^35^ Then, kinetic modeling was performed using the two‐stage Multilinear Reference Tissue Model (MRTM2)^36^ to determine non‐displaceable binding potential (BP_ND_), a metric of binding site density, with cerebellar gray matter as the reference region. Images were projected onto the left and right surfaces and smoothed using a Gaussian Kernel with a full‐width, half‐maximum (FWHM) of 8 mm.

## 3 STATISTICAL ANALYSES

The statistical analyses applied for testing each of our hypotheses were conducted using the R statistical package or MATLAB according to the statistical technique applied.

### 3.1 Comparisons between the TP1 and the TP2

#### 3.1.1 Change in PiB binding

The group analysis general linear model provided by FreeSurfer was applied to investigate the difference in amyloid deposition between TP1 and TP2. Specifically, the one‐factor/two‐level general linear model was applied in each hemisphere. Monte Carlo simulations were implemented to correct for multiple comparisons. The number of permutations was set to 1000. We adjusted the *p*‐values for two hemispheres.

#### 3.1.2 Change in cortical thickness

The longitudinal two‐stage model was used.^32^ The rate of atrophy was calculated through the symmetrized percent change between TP1 and TP2. The symmetrized percent change is the percentage of change corrected for the TP1 and TP2 average thickness, making it more robust than percentage change. Symmetrized percent change maps were smoothed with a 10‐mm FWHM. After that, differences over time were explored using the one‐factor/two‐level general linear model provided by FreeSurfer^37^ in each hemisphere, entering the inter‐scan interval as a covariate. Cluster‐wise correction for multiple comparisons was applied.

#### 3.1.3 PiB binding at TP1 and cortical thickness change at TP2

Cortical thickness estimates and carbon 11–labeled Pittsburgh Compound B ([^11^C]‐PiB) nondisplaceable binding potential (BP_ND_) values from each of the 68 regions of the Desikan‐Killiany atlas were extracted in each participant and time point. For cortical thickness, the values from TP2 were subtracted from those at TP1 and divided by the number of months between scans to determine a measure of the rate of change. A partial Spearman correlation was performed, controlling for age at TP1 in each of the regions where Mak et al.^19^ previously found overlap between amyloid deposition and cortical thickness in a cross‐sectional study. Our aim was to explore if the amount of amyloid at TP1 correlated with the cortical thinning observed in these specific areas between TP1 and TP2.

#### 3.1.4 Multiple regression analyses

Multiple regressions between PiB BP_ND_ and cortical thickness were carried out to study the relationship between these two variables. We explored how change in PiB binding (BP_ND_ (2) − BP_ND_ (1)/time) across the brain was explained by whole‐brain cortical thickness and PiB binding at TP1 using multiple regression analysis, applying the following formula: Whole‐brain PiB change = ß1 Whole‐brain cortical thickness at the TP1 + ß2 Whole‐brain PiB binding at the TP1 + ε.

We further investigated how change in cortical thickness (CTh(2) − CTh(1)/time) across the brain could be explained by whole‐brain cortical thickness and amyloid deposition at TP1, applying the formula: Whole‐brain cortical thickness change = ß1 Whole‐brain cortical thickness at the TP1 + ß2 Whole‐brain PiB deposition at the TP1 +ε.

### 3.2 Time point 3

#### 3.2.1 Comparison between tau deposition and cortical thickness

A Pearson correlation between AV‐1451 BP_ND_ (used to quantify tau deposition) and cortical thickness was calculated across all regions of the Desikan‐Killiany atlas, correcting for multiple corrections (FDR). In addition, a partial Spearman correlation between AV‐1451 BP_ND_ and cortical thickness controlling for age at TP1 was calculated in each of the regions of the Desikan‐Killiany atlas.

## 4 RESULTS

### 4.1 Demographic features

The demographic data are shown in Table 1. Four participants were diagnosed with dementia at TP2: two of them were part of the cognitive decline group at TP1; another one already had dementia; and another one developed dementia during the period between TP1 and TP2 (Table 1). At TP3, when only eight participants returned, there were four participants with no decline, two with cognitive decline, and two with dementia.

### 4.2 Comparisons between TP1 and TP2

#### 4.2.1 PiB binding differences between TP1 and TP2

A voxelwise general linear model analysis was applied between the two time points, correcting for multiple comparisons. No significant differences were found in amyloid burden between TP1 and TP2.

#### 4.2.2 Cortical thickness differences between TP1 and the TP2

**FIGURE 1:**
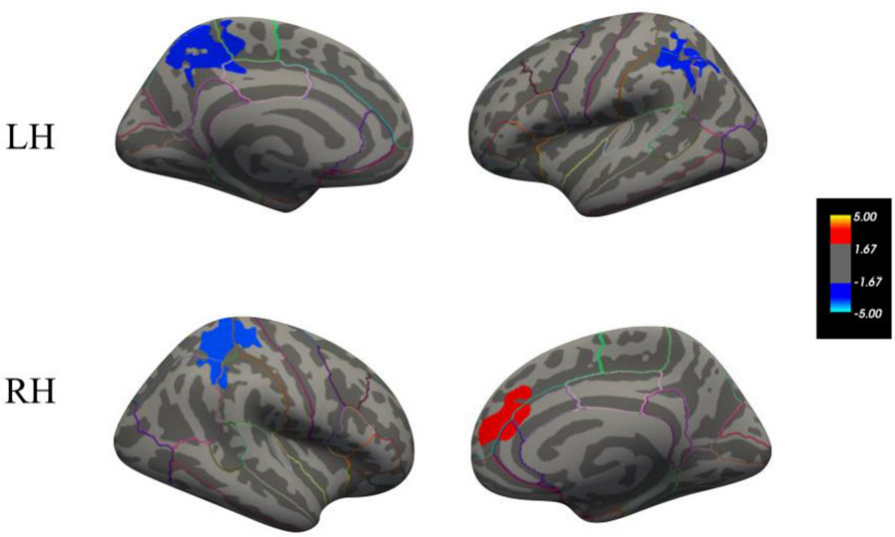
Cortical thickness change between time point 1 (TP1) and time point 2 (TP2) with increases in red and decreases in blue

Clusters of significant atrophy were found in the bilateral superior parietal and the left supramarginal regions (*p* < .001), whereas one cluster of significant cortical thickness increase (*p* < .001) was seen in the right superior frontal region (see Figure 1).

#### 4.2.3 PiB binding at TP1 and cortical thickness change between TP1 and TP2

A partial Spearman correlation controlling for the effect of age was carried out in each of the regions where significant negative correlations between PiB binding (amyloid accumulation) and cortical thinning were found in a previous cross‐sectional study.^19^

**FIGURE 2:**
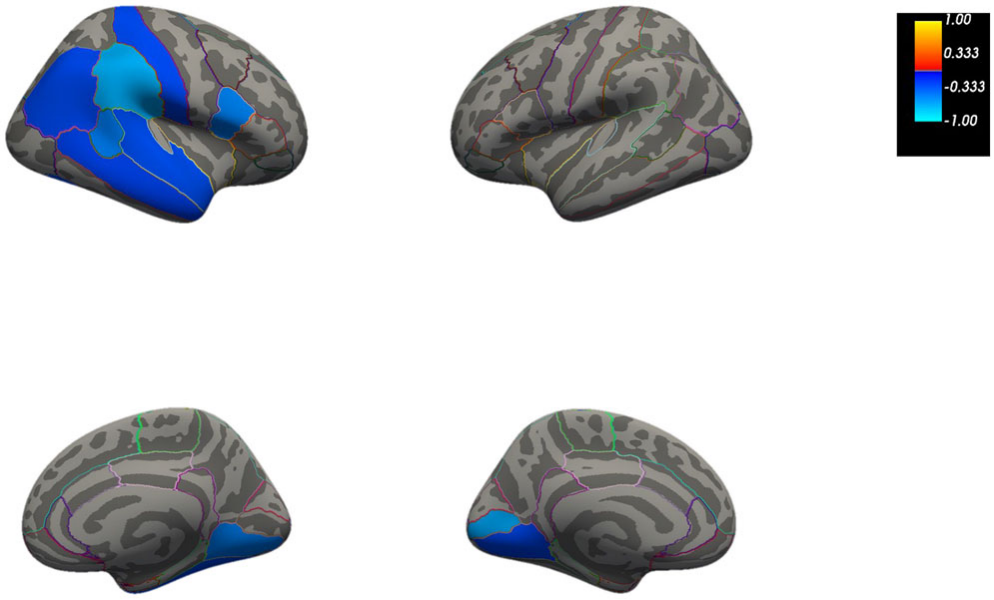
Cortical map showing the regions where carbon 11–labeled Pittsburgh Compound B ([^11^C]‐PiB) nondisplaceable binding potential (BP_ND_) (amyloid deposition) at time point 1 (TP1) was negatively correlated with cortical thickness change between time point 1 (TP1) and time point 2 (TP2). Colors show the strength of the correlation; coldest colors indicate a stronger negative correlation and warmest colors a stronger positive correlation. All correlations were significant at a level of *p* ≤ .05

We observed that PiB BP_ND_ at TP1 was significant and negatively correlated (*p* ≤ .05) with cortical thickness change after 2 years (TP2) in the temporo‐parietal regions (see Figure 2 and Figure S1). Rho coefficients showed a medium to high effect, ranging between −.63 and −.83.

#### 4.2.4 Multiple regression analyses

##### PiB binding change at TP2 explained by amyloid deposition and cortical thickness at TP1

Whole‐brain PiB BP_ND_ change between TP1 and TP2 was explained by PiB BP_N_ at TP1 (ß = 0.006, t = 4.3, *p* < .001), but *not* by cortical thickness at TP1 (ß = −0.000, t = −0.2, *p* > .05). The two predictors explained 6.6% of the variance in PiB binding (*R^2^* ^^= 0.066, *F* (2, 371) = 13.17, *p* < .001). PiB BP_ND_ at TP2 increased 0.006 units for each PiB BP_ND_ unit at TP1.

##### Cortical thickness change at TP2 explained by amyloid deposition and cortical thickness at TP1

Cortical thickness change between TP1 and TP2 was explained by PiB BP_ND_ (*ß* = −0.006, *t* = −5.31, *p* < .001) and cortical thickness (*ß* = −0.002, *t* = −2.49, *p* < .05) at TP1. The results of the regression showed that the two predictors explained 7.1% of the variance in cortical thickness change (*R^2^* ^^= 0.071, *F* (2, 371) = 14.12, *p* < .001). A PiB BP_ND_ unit at the TP1 explained a reduction of 0.06% in cortical thickness (mm).

### 4.3 Time point 3

#### 4.3.1 Correlation between tau deposition and cortical thickness at time‐point 3

**FIGURE 3:**
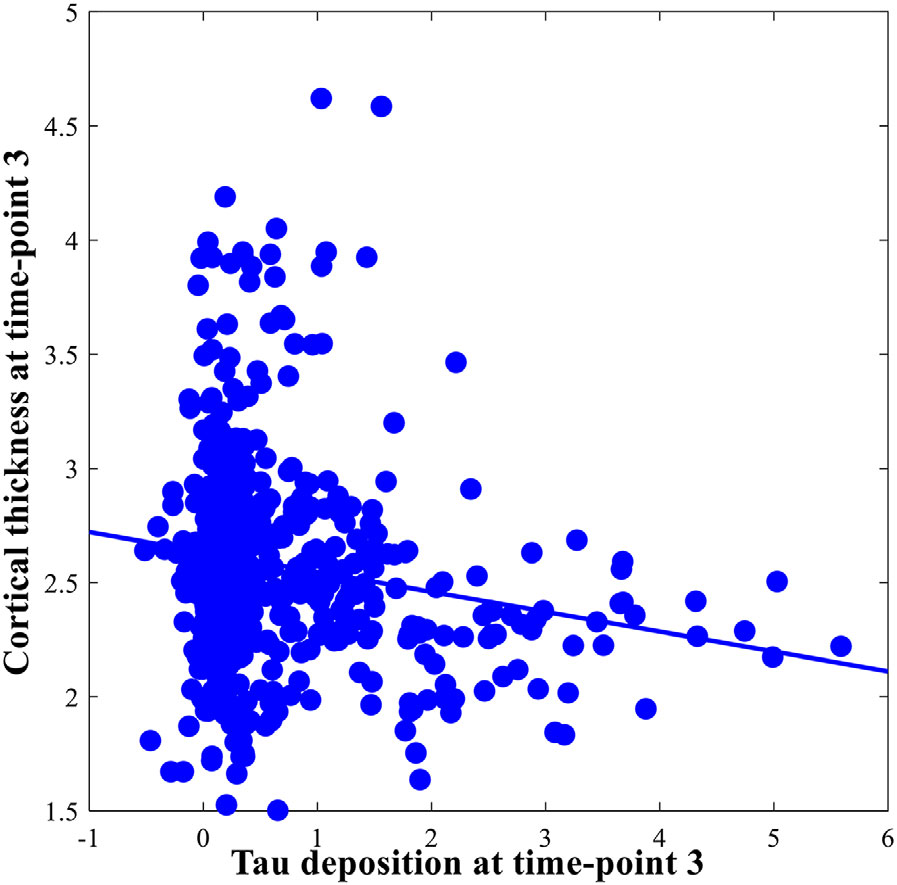
Pearson correlation between AV‐1451 nondisplaceable binding potential (BP_ND_) (tau deposition) and cortical thickness at time‐point 3. All correlations were significant at a level of *p* ≤ .05 and corrected for multiple comparisons applying the false discovery rate (FDR) method.

A Pearson correlation between AV‐1451 BP_ND_ (tau deposition) and cortical thickness was calculated across all regions of the Desikan‐Killiany atlas, correcting for multiple corrections (FDR). It revealed a significant negative correlation (see Figure 3) between AV‐1451 BP_ND_ and cortical thickness (*r* = −.2, *p* < .001).

**FIGURE 4:**
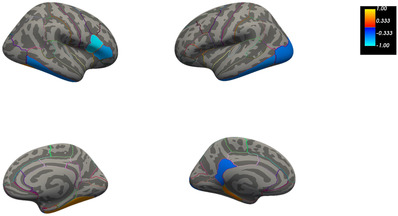
Cortical map showing those areas where the correlation between AV‐1451 nondisplaceable binding potential (BP_ND_) (tau deposition) and cortical thickness at time point 3 was significant. Colors show the strength of the correlation; coldest colors indicate a stronger negative correlation and warmest colors a stronger positive correlation. All correlations were significant at a level of *p* ≤ .05

In addition, a partial Spearman correlation between AV‐1451 BP_ND_ and cortical thickness was calculated in each of the regions of the Desikan‐Killiany atlas, controlling for the effect of age. The results showed significant negative correlations in the left and right inferior temporal, left isthmus cingulate, left lateral occipital regions, right pars opercularis and triangularis regions (see Figure 4 and Figure S2). All these regions had an rho value between −.77 and −.94 (*p* *< *.05), despite the small sample size, which reflects a medium to large effect size. There were positive significant correlations (*p* *< *.05) in the left parahippocampal and right fusiform regions with rho values of .8, indicating a large effect size (see Figure 4 and Figure S2).

## 5 DISCUSSION

In this longitudinal study on DS, we first explored the pattern of change of amyloid deposition and cortical thickness after an average period of 2 years. Second, we investigated the regions affected by tau in a subgroup of eight patients returning for scanning a further 2 years later, almost 5 years after the first visit. We were particularly interested in studying whether regions with significant tau accumulation overlapped with those having cortical atrophy.

First, we found that after 2 years, whole‐brain amyloid deposition did not change significantly. Second, there was pronounced cortical thinning over this period in the temporo‐parietal cortices, although thickening was observed in the frontal cortex. Third, amyloid deposition at TP1 negatively correlated with the *change* in cortical thickness over the following 2 years. There was a complete overlap between amyloid deposition at TP1 and cortical thinning in temporo‐parietal regions 2 years later. Fourth, multiple regression analyses demonstrated that the rate of cortical thinning was explained by the level of amyloid deposition at TP1. Of note was the observation that cortical thickness at TP1 did not explain the change in amyloid deposition. Finally, and more importantly, tau deposition was negatively correlated with cortical thinning, both across the brain and in specific regions of the brain, showing the relationship typically found in sporadic and autosomal AD.

Of interest, cortical thickness decreased significantly after 2 years in several brain areas, but amyloid deposition did not increase. Jack et al.^38^ explained it as a dissociation between ongoing amyloid and atrophy progression, indicating that amyloid reaches an early plateau during the course of the disease,^39^ whereas cortical thinning continues to progress over time.

Differences found in cortical thickness, with a thinner cortex in the posterior‐temporal areas and a thicker cortex in frontal areas, might indicate that each of these regions is in a different stage in the process of cortical neurodegeneration. As Annus et al.^8^ showed, the posterior lobes are the first regions presenting with atrophy, which is corroborated by our results. However, the frontal lobe may still be at an earlier stage of the disease, and other events, such as inflammation, might be taking place as suggested by Fortea et al.^40^ These authors observed that presymptomatic ADAD participants with abnormal CSF levels of amyloid, but normal CSF levels of phosphorylated tau (p‐tau) had an increase in cortical thickness. Montal et al.^41^ confirmed this two‐phase phenomenon, explaining it as a biphasic model according to which there is an increase in cortical thickness when the CSF amyloid levels decrease, but the CSF p‐tau is normal; and later as the disease progresses and the CSF tau levels increase, the cortex becomes thinner. This might be applied to our data, in which each participant might be in a different phase of disease progression, and therefore, have different levels of CSF amyloid and tau. The amount and distribution of amyloid and tau in the brain will also differ according to disease stage. Further analyses using CSF along with plasma amyloid and tau biomarkers will be required in our sample to investigate the interaction of tau with coexisting amyloid burden on the progression of brain atrophy. The data and analyses conducted in this study do not currently answer this question.

Regarding the negative relationship found for some regions between high levels of amyloid deposition at TP1 and cortical thinning between TP1 and TP2, these results are important because they demonstrate that the degree of amyloid accumulation in the brain at TP1 is highly associated with the change in cortical thickness over the following 2 years. Nevertheless, this overlap does not imply causality, but rather suggests that a higher amount of amyloid accumulation triggers a cascade of changes of disease‐causing processes such as inflammation and tau‐tangle formation that eventually lead to dementia.^38^

Tau starts aggregating in the medial temporal lobe following Aβ deposition in the medial parietal lobe and striatum.^11^ Unlike amyloid, it has been argued that the deposition of tau in the brain participates in the process of cortical degeneration.^20^ For this reason, regions showing high levels of tau accumulation also present significant hypometabolism and atrophy.^42^ In DS, however, there are not many studies investigating whether the same regions where tau is accumulated are the ones showing cortical thinning. In our study, we have found that regions in the medial and basal temporal lobe that usually accumulate tau at Braak stages III to V also showed cortical atrophy.

In sum, whole‐brain amyloid burden at TP1 was highly associated with the progression of cortical thinning over the next 2 years. At the regional level, tempo‐parietal regions showed negative correlations between these two biomarkers. Global levels of tau deposition were negatively correlated with cortical atrophy, with high correlations found in regions where tau usually accumulates at later Braak stages.^24^

## CONFLICTS OF INTEREST

All authors declare no competing interests. This research was funded by different grants from the Medical Research Council (grant ID number: 98480), the Alzheimer's Research UK (grant ID number: ARUK‐PG2015‐23), and the National Institutes of Health (NIH) (grant ID number: U01AG051406‐01 Neurodegeneration in Aging Down Syndrome [NiAD]). Additional support came from the National Institute for Health Research (NIHR) Cambridge Biomedical Research Centre, the NIHR Collaborations in Leadership for Applied Health Research and Care (CLAHRC) for the East of England, the NIHR Cambridge Dementia Biomedical Research Unit, the Down Syndrome Association, and the Health Foundation. *Dr Concepcion Padilla* is currently funded by a Sara Borrell Postdoctoral Fellowship (CD20/00133, Carlos III Health Institute) and she was previously paid from a grant awarded to the Cambridge Intellectual and Developmental Disabilities Research Group by the Alzheimer Research UK (grant ID number: ARUK‐PG2015‐23). She has been paid as a lecturer at the Open University of Catalonia and at the Rioja International University, Spain. She received travel grants for attending conferences from the Guarantors of Brain and Alzheimer Research UK. She has nothing else to disclose. *Mr Victor Montal* is currently receiving a salary from the Memory Unit and the Biomedical Research Institute Sant Pau (IIB Sant Pau), Neurology Department, Hospital de la Santa Creu i Sant Pau, Barcelona, Spain. He received consulting fees from personal consultancy related to a R21 grant of Dr Vannini at the Mass General Hospital. He has nothing else to disclose. *Madeleine J. Walpert* is currently paid as a policy advisor at Alzheimer's Research UK and previously received a postdoctoral fellowship as a research associate by Alzheimer's Research UK. She has nothing else to disclose. *Young T. Hong* receives his salary from the University of Cambridge. He is also supported by the United States (US) National Institutes of Health as part of the NiAD—Cambridge team. He has nothing else to disclose. *Tim D. Fryer* receives his salary from the University of Cambridge. He has nothing else to disclose. *Dr Jonathan P. Coles* is supported by the NIHR Cambridge Biomedical Research Centre. He has nothing else to disclose. *Dr Franklin I. Aigbirhio* receives his salary from the University of Cambridge. He was awarded with an Engineering and Physical Sciences Research Council (EPSRC) Project grant as a co‐applicant, although only the principal applicant was funded. He has led the Dementia Platform UK Imaging Network, receiving no payments. He has nothing else to disclose. *Dr Sigan L. Hartley* received a grant from the US National Institutes of Health, although it is not related to this study. She has nothing else to disclose. *Dr Ann D. Cohen* was awarded with the grants R01 AG052446, P01 AG025204, R01 AG063525, U01 AG051197, U01 AG051406, P30 AG066468, U19 AG068054, RF1 AG052525, R01 AG056351, and UL1 TR001857‐04S1; however, all of them were made to the University of Pittsburgh. She has been involved in the International Society to Advance Alzheimer's Research and Treatment (ISTAART) advisory board. She has nothing else to disclose. *Dr Dana L. Tudorascu* is paid by the University of California Irvine. She has participated on a Data Safety Monitoring Board (DSMB), receiving no payments. She has nothing else to disclose. *Dr Bradley T. Christian*: The NIH provided support to his institution for this research. He is involved and receives funding from the following projects: NIH/National Institute on Aging (NIA) PET Imaging agents for a4b2 Nicotinic Receptors, P30 AG062715 NIH/NIA Wisconsin Alzheimer's Disease Research Center, U54 HD090256 NIH/NICHD The Wisconsin Intellectual and Developmental Disabilities Research Center, R01 AG021155 NIH/NIA The longitudinal course of neural function and amyloid in people at risk for Alzheimer's Disease (AD), R01 AG027161‐11 NIH/NIA Wisconsin Registry for Alzheimer Prevention, R01 AG059312 Algebraic Formulations for Characterizing Structural Brain Connectivity Changes and Pathology Transmission Networks in Preclinical Alzheimer's Disease, R01 AG062167 NIH/NIA Longitudinal Investigation of Cardiorespiratory Fitness and AD Biomarkers in an At‐Risk Cohort, R01 AG063752‐01 NIH/NIA Statistical methods to improve reproducibility and reduce technical variability in heterogeneous multimodal neuroimaging studies of Alzheimer's Disease, R61 HD100973‐01 NIH/NIA Clinical trials to prevent Alzheimer's Disease in Down Syndrome, R01 AG060737‐03S1 NIH/NIA Wisconsin Longitudinal Study–Initial Lifetime's Impact on Alzheimer's Disease and Related Dementias (WLSILIAD Study), R01 AG70028 NIH INCLUDE Lifestyle Risk and Resiliency Factors and Alzheimer's Disease in Down syndrome, and R01 AG058533‐01A1 Health and Aging Brain among Latino Elders (HABLE‐AT[N]). He has also participated on a DSMB for the UO1 AG15001 NCE NIH/NICHD/NIA Neurodegeneration in Aging Down Syndrome (NiAD) project, receiving no payments for his involvement. He has received Cerveau and AVID, that was made to the institution. He has nothing else to disclose. *Dr Benjamin L. Handen* receives funding from the National Institute on Aging and the National Institute of Child Development, Autism Speaks, and Roche. He receives book royalties from Oxford University Press. He is also chair of a DSMB for a Department. of Defense funded study in autism. He has nothing else to disclose. *Dr William E. Klunk* is supported by the NIH/NIA. He is involved on a DSMB for Biogen. He has nothing else to disclose. *Prof Anthony J. Holland* was awarded with a grant from the Alzheimer's Research UK. He has been the President of the International Prader Willi Syndrome Organization. This was a volunteer post, and no payments were made. He has nothing else to disclose. *Dr Shahid H. Zaman* is paid by the UK National Health Service (NHS) and has received funding to attend meetings or conferences by the NIH and the Collaborations in Leadership for Applied Health Research and Care (CLAHRC) for the East of England. He has attended a DSMB meeting as part of a NIH‐funded on‐going research project. He has nothing else to disclose.

## Supporting information

Supporting information

Supporting information
