# Supplementary material for: Cortical atrophy and amyloid and tau deposition in Down syndrome: A longitudinal study
Source: Alzheimers Dement (Amst). 2022 Apr 1;14(1):e12288. doi: 10.1002/dad2.12288 (PMC8974205; doi:10.1002/dad2.12288)
Supplement: Supplementary file 1 — Supporting information [file DAD2-14-e12288-s001.pdf]

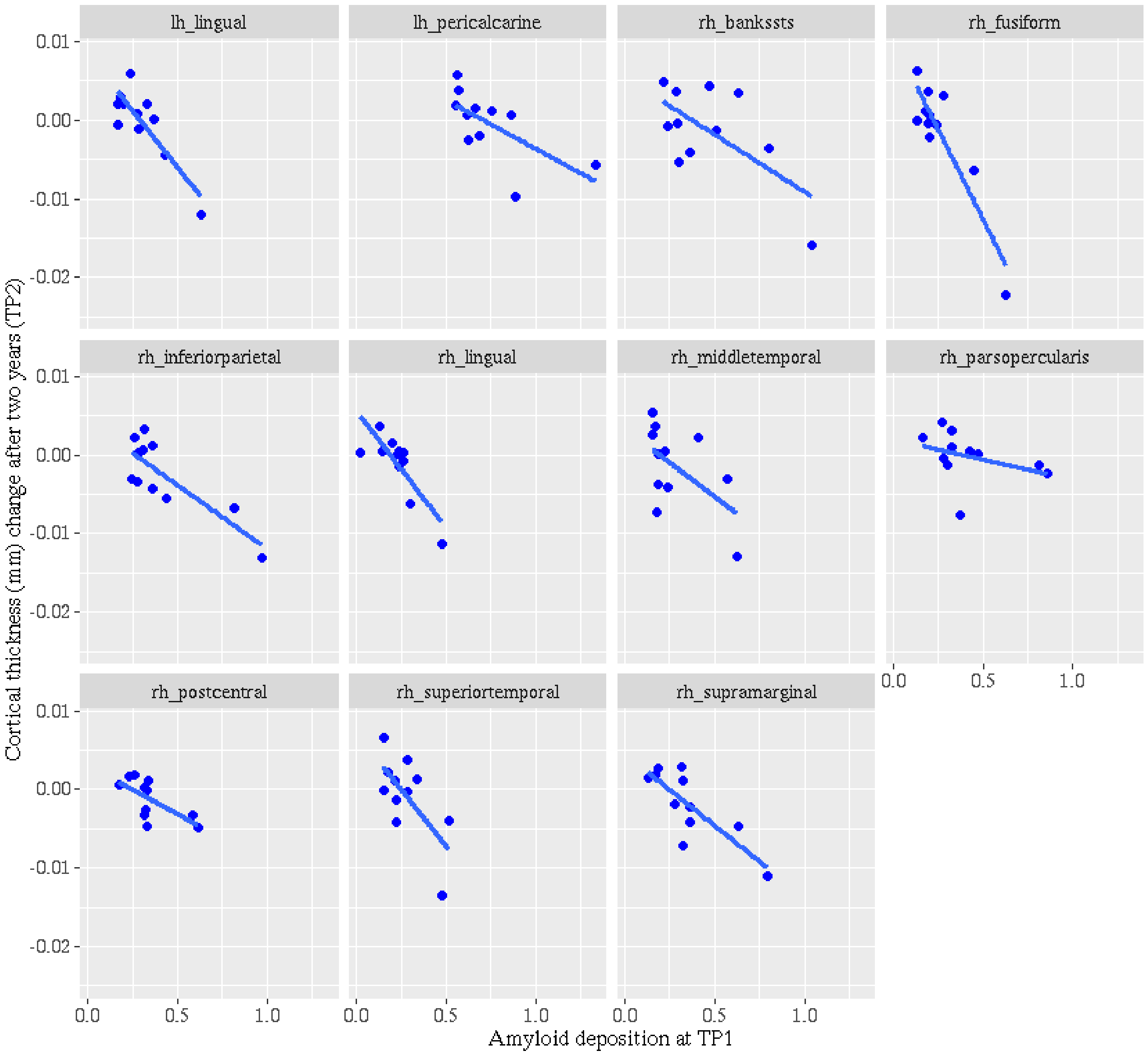

Supplementary Figure 1. Partial Spearman correlation controlling for the effect of age between PiB binding (BP_{ND}) at TP1 and cortical thickness change between TP1 and TP2 in each of the regions found with significant negative correlations.
