# Supplementary material for: Cortical atrophy and amyloid and tau deposition in Down syndrome: A longitudinal study
Source: Alzheimers Dement (Amst). 2022 Apr 1;14(1):e12288. doi: 10.1002/dad2.12288 (PMC8974205; doi:10.1002/dad2.12288)
Supplement: Supplementary file 2 — Supporting information [file DAD2-14-e12288-s002.pdf]

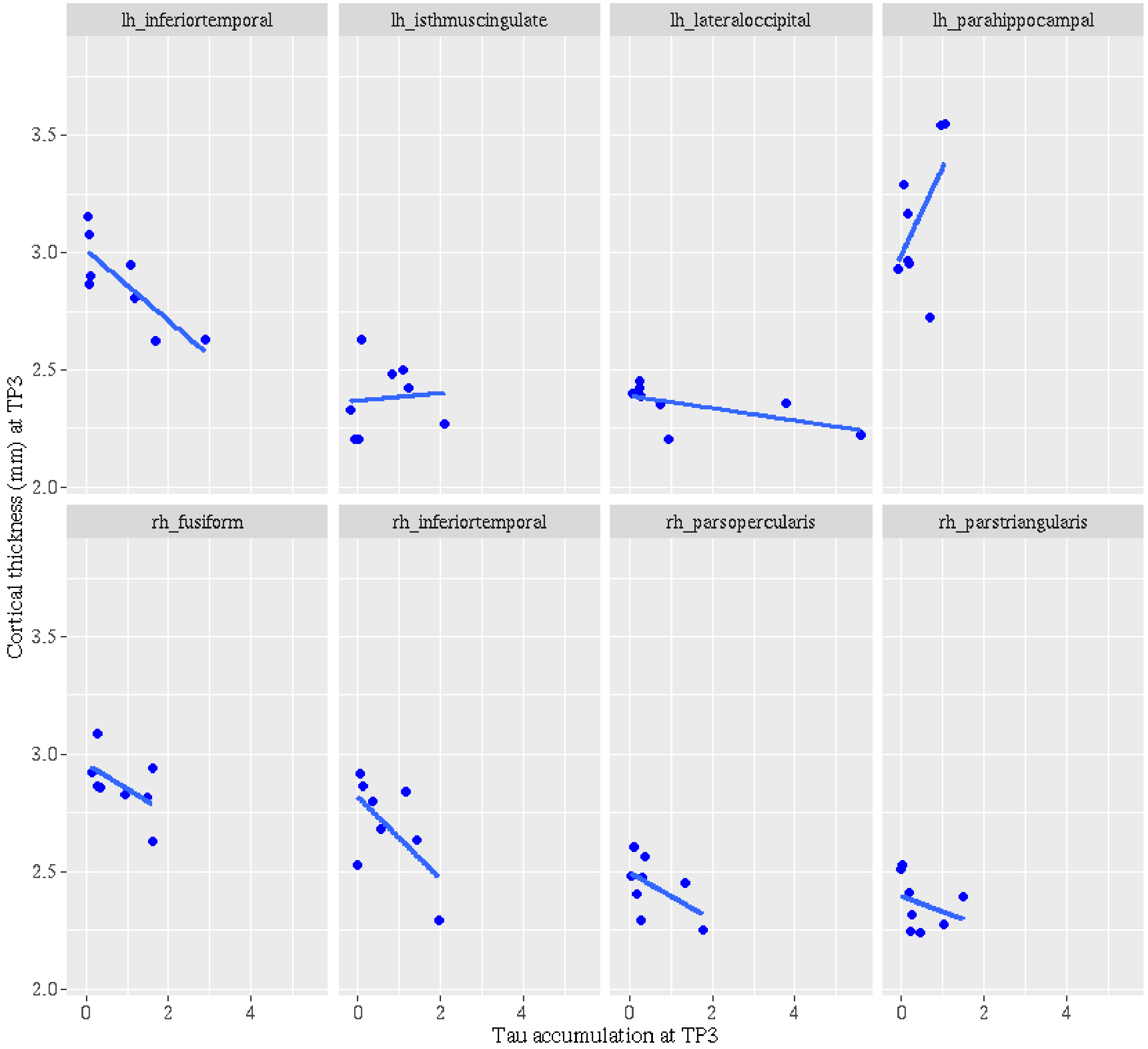

Supplementary Figure 2. Partial Spearman correlation controlling for the effect of age between AV-1451 BPND (tau deposition) and cortical thickness (mm) at time-point 3 (TP3). All regions showed significant correlations ($p < 0.05$).
